# Health-Related Quality of Life in Elderly Patients with Pacemakers

**DOI:** 10.21470/1678-9741-2020-0522

**Published:** 2021

**Authors:** Natielly Aleixo Inácio, Manoel Muniz Neto, Antônio da Silva Menezes Junior, Joaquim Ferreira Fernandes, Vinícius Araújo Barbosa, Tiago de Almeida Laranjeira, Marcos Arruda

**Affiliations:** 1 School of Medical, Pharmaceutical and Biomedical Sciences, Pontifical Catholic University of Goiás (PUC-GO), Goiânia, GO, Brazil.; 2 Department of Psychoanalysis, Vincentian College, Curitiba, PR, Brazil.

**Keywords:** Quality of Life, Surveys and Questionnaires, Pacemaker, Artificial, Aged, Perception

## Abstract

**Introduction:**

Cardiac pacemaker (PM) therapy is of paramount importance. PM use increases with age, with an estimated increased use of 70% to 80% in patients over 65 years. This study evaluated the perception of the health-related quality of life (HRQoL) of elderly patients with PM, comparing them with patients without PM, by applying two quality of life questionnaires: EuroQoL 5-dimensions (EQ-5D) and 36-Item Short Form Health Survey (SF-36).

**Methods:**

This study included elderly patients divided into a group with PM and another without PM. Information on HRQoL was obtained using the EQ-5D and SF-36 questionnaires.

**Results:**

The study involved 104 elderly patients with PM and 150 without PM. The distribution of responses to the EQ-5D was similar between groups. Statistical differences within the gender variable in the group of elderly people with PM were significant for the mobility, habitual activities, and anxiety/depression domains and for the average EQoL utility score and visual analogue scale (EQ-VAS). Elderly patients with PM presented significant differences between New York Heart Association classes 1 and 2 for the mobility domain and EQ-VAS, while those evaluated through SF-36 presented higher averages in vitality, general health status, and pain. However, a different analysis was observed in the physical aspect domain.

**Conclusion:**

The SF-36 demonstrated that elderly patients with PM had an HRQoL similar to or greater than those without PM. However, the results of the EQ-5D did not show significant differences regarding the implantation of PM and HRQoL between the two groups of elderly individuals in the study.

**Table:** 

Abbreviations, acronyms & symbols	
ANOVA	= Analysis of variance
DM	= Diabetes mellitus
EQ-5D	= EuroQoL 5-dimensions
EQ-VAS	= EuroQol Visual Analogue Scale
HRQoL	= Health-related quality of life
HT	= Hypertension
MCS	= Mental component score
NYHA	= New York Heart Association
PCS	= Physical component score
PM	= Pacemaker
SF-36	= 36-Item Short Form Health Survey
TTO	= Time trade-off

## INTRODUCTION

The use of cardiac pacemakers (PM) as a therapeutic modality for the treatment of abnormalities in the electrical conduction of the heart is a challenge that seeks to add quality of life to the prognosis of patients with heart disease ^[1]^. PM therapy has a lasting impact on the individual's life, since its implantation along with an intervention is a continuous treatment requiring successive checks of the device and, if necessary, surgeries for PM replacement ^[2]^.

Several PM implants have been performed worldwide, and particularly in Brazil, according to data from the Brazilian Registry of Pacemakers, Defibrillators and Cardiac Resynchronizers, more than 306 thousand device implantation surgeries were registered from 2000 to 2014 ^[3]^. The frequency of use of these devices increases with age, with an estimated increased use of 70% to 80% in patients over 65 years of age ^[4]^. Therefore, the assessment of the health-related quality of life (HRQoL) has become critical in the management of patients with heart disease. The patient’s perception of their own health status and quality of life emerges as a guide in interpreting and analyzing the effectiveness of the treatment received ^[1]^.

According to the World Health Organization, quality of life is the "individual's perception of his or her position in life in the context of the culture and value system in which he or she lives and in relation to his or her goals, expectations, standards, and concerns" ^[5]^. Several instruments were developed for the evaluation of HRQoL among generic and specific questionnaires. Among these, the 36-Item Short Form Health Survey (SF-36) and EuroQoL 5-dimensions (EQ-5D), which provide a descriptive profile of health status and a general utility score of HRQoL, stand out ^[6]^.

A study conducted with 501 patients in the Netherlands (2008) quantifying the average differences between HRQoL before PM implantation and after one year, assessed using the SF-36 and EQ-5D questionnaires and AQUAREL (specific for PM patients), observed that the HRQoL increased in the first year after the implant in most patients who received PM ^[7]^. Another study conducted with 107 patients in Brazil (2013) showed a negative correlation between quality of life and functional class and pointed out that the more advanced the patient’s age, the worse the HRQoL in functional capacity and class ^[8]^.

The EQ-5D and SF-36 instruments are, therefore, the two generic questionnaires of HRQoL used worldwide ^[9]^. SF-36 is the main generic questionnaire used to evaluate HRQoL in patients using PM ^[10]^. The EQ-5D has many applications in the cost-benefit evaluation of health treatments ^[11]^. However, in the literature, no studies have reported results on the use of the EQ-5D and SF-36 questionnaires to evaluate the specific HRQoL for elderly people using PM in the same study.

The objective of this study was, therefore, to evaluate the perception of HRQoL in elderly patients with PM compared to those without PM, by applying the two generic EQ-5D and SF-36 questionnaires. At the end of the study, we intended to observe the similarities and differences in the results on the HRQoL of the EQ-5D and SF-36 questionnaires applied to both groups of elderly patients.

## METHODS

The study protocol was approved by the Research Ethics Committee of the Pontifical Catholic University of Goiás, according to resolution 466/12 of the Ministry of Health. All patients were informed about the study and all signed the informed consent form.

A cross-sectional, quantitative, and analytical observational study was conducted with a group of elderly patients with PM and a group of patients without PM randomly selected at a cardiology clinic, Stimulocoeur Assessment and Research, in Goiânia, Brazil. Data were collected from November 2018 to January 2019. The sample size was defined in 254 elderly individuals, 104 with PM and 150 without PM.

Elderly patients of both genders, aged 60 years or older, without PM or with PM implant for more than two years, clinically stable, and with New York Heart Association (NYHA) classes 1 and 2 participated in the study. 

Demographic data were collected through a questionnaire developed by the researchers according to the literature ^[9,12]^ containing personal data such as age, gender, education, comorbidities (hypertension, diabetes mellitus, chronic kidney disease, and chronic obstructive pulmonary disease), whether they had PM and the duration of device implantation, and NYHA functional classification. Information on HRQoL was collected through two generic questionnaires: EQ-5D and SF-36. The research was performed orally by the researchers.

### Description of Instruments

#### EQ-5D

The EQ-5D is a generic questionnaire on HRQoL, created in 1990 by the EuroQol group, widely used in clinical, observational, economic, population studies and other health research ^[13]^.

The instrument consists of a descriptive system in the form of a questionnaire and a visual analog scale (EQ-VAS). The first part is a standardized measure of health status containing five domains of the daily quality of life, such as mobility, personal care, habitual activities, pain/discomfort, and anxiety/depression, with three levels of severity in each domain (without problems, some problems, and serious problems), generating 243 possible health states by combining the value given in each of the five dimensions. The EQ-VAS evaluates the individual's perception of his/her current general health status, using a score scale between 0 (worst imaginable health status ) and 100 (best imaginable health status) ^[9]^.

#### EQ-5D Score

The HRQoL scores cannot be directly obtained through the EQ-5D questionnaire responses. Thus, it was necessary to use a time trade-off (TTO) model to transform the measures into utility health scores. In this study, a Brazilian TTO model was used, which was performed through a multicentric cross-sectional study ^[14]^.

The utility value (EQoL) for health status 11111, classified as "without problems" in all five domains, was set at 1. To calculate the other predicted values for any health condition other than 11111, the following algorithm was used: 0,851+ (-0,120 * M2) + (-0,363 * M3) + (-0,112 * CP2) + (-0,218 * CP3) + (-0,097 * AH2) + (-0,184 * AH3) + (-0,064 * DD2) + (-0,168 * DD3) + (-0,050 * AD2) + (-0,095 * AD3). M2, CP2, AH2, DD2, and AD2 represent the second level ("some problems") of the mobility, personal care, habitual activities, pain/discomfort, and anxiety/depression domains, respectively. M3, CP3, AH3, DD3, and AD3 represent the third level ("serious problems") of mobility, personal care, habitual activities, pain/discomfort, and anxiety/depression domains, respectively ^[14]^.

#### SF-36

The SF-36 is a generic multidimensional quality-of-life assessment instrument consisting of 36 items, encompassed in eight domains, including functional capacity, physical aspects, pain, general health, vitality, social aspects, emotional aspects, and mental health. It has a final score of 0 (worst general health status) to 100 (best health status) for each of the eight domains ^[12]^. Finally, these domains are grouped into two summary measures, physical component score (PCS) and mental component score (MCS). PCS is calculated based on a combination of the domains of functional capacity, physical aspects, pain, and general health status, while MCS is based on a combination of the domains of vitality, social aspects, emotional aspects, and mental health ^[15]^.

#### SF-36 Score

The scores of the eight SF-36 domains as well as the PCS and MCS summary measure scores were calculated according to the authors’ guidelines of the SF-36 questionnaire ^[16]^.

#### Statistical Analysis

Data were tabulated and presented in tables, percentages, and measures of central tendency and dispersion. To compare the quantitative variables and the categorical variables between the groups of elderly people with and without PM, Student’ s t-test, analysis of variance (ANOVA), and Pearson’s chi-square test were used. The level of significance was set at 5%.

## RESULTS

The study included a total of 254 elderly people, 150 without PM, with an average age of 69.02 years (+/-7.37 years), and 104 with PM, with an average age of 72.26 years (+/-9.21 years), and an average of 6.52 years (+/-4.75 years) after PM implantation.

The general profile of the elderly in the two groups analyzed was predominantly female (61%), with a prevalent age range of 60 to 69 years (53.94%). Half of the elderly interviewed had elementary school education level (50%) and had hypertension as the most prevalent comorbidity (51.6%) (Table 1).

**Table 1:** General and clinical characteristics of the elderly patient population (n=254).

Variables		n	Distribution (%)
Gender	Female	155	61
	Male	99	39
Age (years)	60-69	137	53.94
	70-79	74	29.14
	80-89	40	15.74
	>90	3	1.18
Education level	Illiterate	27	10.6
	Elementary school	127	50
	High school	62	24.4
	Higher education	38	14.9
Comorbidities	Hypertension	131	51.6
	Diabetes mellitus	48	18.9
	Chronic kidney disease	3	1.2
Functional classification (NYHA)^*^	Class 1	43	16.9
	Class 2	13	5.1
Time of PM implantation (years)	Average (SD)	6.52 (4.75)	
	Minimum-maximum	3 months-25 years	

NYHA=New York Heart Association; SD=standard deviation

### EQ-5D

The distribution of responses to the five domains of the EQ-5D was similar between the two groups analyzed, with the "no problem" response being the median of the mobility, personal care, habitual activities, and anxiety/depression domains, and "some problems" the median of the pain/discomfort domain.

The elderly without PM were divided into three groups according to their responses to the five domains analyzed: “no problem”, “some problems”, and “serious problems”. Mobility was not affected in 101 patients without PM; however, 48 elderly patients reported some problems and 1 had serious problems. Regarding the personal care domain, there was no impairment for 132 elderly patients without PM, while 18 reported some problems, and none of them had serious problems. A total of 98 elderly people did not have impaired habitual activities, 51 had some problems, and 1 had serious problems. In relation to anxiety/depression, 81 patients without PM had no problems, 59 had some problems, and 10 had serious problems. Concerning the pain/discomfort domain, there were 34 elderly people without problems, 105 with some problems, and 11 with serious problems.

In the same way as the group without PM, patients with PM were divided according to their responses to the five domains, and regarding the mobility domain, 86 elderly people had no problems, 18 had some problems, and none of them had serious problems. Personal care was not affected in 99 patients, while 5 others had some problems and none had serious problems. There was no impairment of habitual activities for 59 patients, but 43 reported some problems, and 2 of them had serious problems. In relation to anxiety/depression, 41 patients with PM did not develop any problems, 55 had some problems, and 8 had serious problems. Finally, concerning the pain/discomfort domain, 52 patients had no problems, 43 had some problems, and 9 had serious problems.

The analysis of the EQ-VAS scores did not show a statistically significant difference between the elderly groups (P=0.152). The elderly without PM had an average score of 76.37 (+/-15.47), and those with PM had an average score of 78.96 (+/-11.95), and both had medians equal to 80. 

The study showed only a small portion of the 243 possible utility vectors for the EQ-5D. Elderly patients without PM presented 31 vectors, of which seven represented 70.7% of the survey responses. The elderly with PM presented 30 vectors, of which seven represented 68.3% of the total responses of the interviewees (Tables 2 and 3).

**Table 2:** Frequency of EQ-5D health status of patients without pacemaker.

Vectors	n	% of total	Accumulated (%)
11111	23	15.30	15.30
11121	24	16.00	31.30
11122	23	15.30	46.70
11222	8	5.30	52.00
21122	8	5.30	57.30
21221	8	5.30	62.70
22221	12	8.00	70.70
24 others	44	29.30	100.00
Total	150	100.00%	

**Table 3:** Frequency of EQ-5D health status of elderly patients with a pacemaker.

Vectors	n	% of total	Accumulated (%)
11111	16	15.40	15.40
11112	11	10.60	26.00
11121	14	13.50	39.40
11122	10	9.60	49.00
11211	7	6.70	55.80
11221	7	6.70	62.50
21222	6	5.80	68.30
23 others	33	31.70	100.00
Total	104	100.00%	

The percentage of elderly with PM who answered 2 ("some problems") or 3 ("serious problems") in the EQ-5D, according to the demographic variables, is shown in Table 4. 

**Table 4:** Responses to the EQ-5D according to the demographic variables of elderly people with a pacemaker (PM).

Variables	n	% of elderly people with PM who reported having some problems or serious problems										Average EQoL (SD)	*P*	Average EQ-VAS (SD)	*P*
		M	*P*	PC	*P*	HA	*P*	PD	*P*	AD	*P*				
**Age (years)**			0.478		0.867		0.716		0.099		0.978		0.593		0.523
60-79	80	14.42%		3.85%		33.65%		43.27%		38.46%		0.734 (0.163)		79.53 (10.98)	
80-100	24	2.88%		0.96%		19.23%		34.62%		11.54%		0.713 (0.144)		77.04 (14.84)	
**Gender**			0.0001		0.06		0.00		0.00		0.0002		0.001		0.016
Female	62	17.30%		3.85%		2.3%		41.35%		39.43%		0.689 (0.170)		76.62 (13.03)	
Male	42	0.00%		1.93%		16.35%		19.23%		10.58%		0.786 (0.118)		82.35 (9.30)	
**Education level**			0.769		0.919		0.824		0.708		0.399		0.423		0.804
Illiterate	17	2.89%		0.96%		7.70%		11.54%		7.70%		0.702 (0.131)		77.35 (12.24)	
Elementary school	54	10.58%		1.92%		21.15%		30.77%		23.07%		0.743 (0.166)		78.89 (11.34)	
High school	21	2.89%		0.96%		10.58%		15.39%		12.50%		0.691 (0.150)		81 (11.69)	
Higher education	12	0.97%		0.96%		3.85%		2.89%		6.73%		0.768 (0.170)		77.96 (15.45)	
**Time of PM implantation**			0.18		0.825		0,133		0.504		0.975		0.148		0.065
>3 months-1 year	14	2.89%		0.96%		4.81%		7.70%		3.85%		0.741 (0.191)		81.39 (9.67)	
1-5 years	35	8.65%		1.92%		18.27%		18.27%		13.46%		0.686 (0.153)		75.14 (13.75)	
>5 years	55	5.77%		1.92%		20.19%		34.61%		32.70%		0.753 (0.150)		80.76 (10.79)	
**Comorbidities**															
Hypertension	53	12.50%	0.04	1.92%	0.619	21.15%	0,72	37.50%	0.466	28.85%	0.199	0.697 (0.132)	0.037	76.92 (13.35)	0.227
Diabetes	22	3.85%	0.904	2.88%	0.03	9.61%	0,41	13.46%	0.109	10.58%	0.656	0.707 (0.169)	0.486	76.13 (14.63)	0.214
**Functional classification (NYHA)**			0.006		0.257		0,202		0.488		0.056		0.064		0.0002
Class 1	28	6.73%		1.92%		11.54%		18.27%		16.35%		0.706 (0.170)		75.07 (10.74)	
Class 2	6	3.85%		0.96%		3.85%		1.92%		4.80%		0.603 (0.255)		64.34 (15.73)	

AD=anxiety/depression; HA=habitual activities; M=mobility; NYHA=New York Heart Association; PC=personal care; PD=pain/discomfort; SD=standard deviation

Statistical differences between the gender variable in the group of elderly people with PM were significant for the domains of mobility (*P*=0.0001), habitual activities (*P*=0.000), and anxiety/depression (*P*=0.0002) as well as for the average EQoL utility score (*P*=0.001) and EQ-VAS scores (*P*=0.016). Those with arterial hypertension had lower values in the mobility domain (*P*=0.04) and in the average EQoL score (*P*=0.037) compared to the same group of patients with PM. Differences between NYHA functional classes 1 and 2 were significant for the mobility domain (*P*=0.006) and EQ-VAS scores (*P*=0.0002) (Table 4).

### SF-36

The average PCS score for the elderly patients without PM was 66.7 (+/-16.58), with a median of 65.375, and the average MCS score was 67.7 (+/-18.76), with a median of 71. For patients with PM, the average PCS score was 70.29 (+/-20.67), with a median of 70.875, and the average MCS score was 70.25 (+/-22.45), with a median of 73.

In each of the eight SF-36 domains, the average and standard deviation values were calculated for the two groups of elderly people analyzed. Patients with PM presented higher statistically significant averages in the domains of vitality (*P*=0.00), general health status (*P*=0.00), and pain (*P*=0.00) in comparison to those without PM. Different analyzes were observed in the physical aspects’ domain, in which patients with PM presented a lower average (*P*=0.001) than those without PM (Table 5). 

**Table 5:** Average scores and standard deviation of SF-36 domains for elderly patients with and without PM.

Variable	n	MH	P	EA	P	SA	P	VT	P	GHS	P	Pain	P	PA	P	FC	P
Elderly people with PM	104	73.27 (22.23)	0.85	58.59 (42.99)	0.23	74.20 (27.20)	0.13	74.95 (21.98)	0.00	78.70 (16.76)	0.00	80.55 (36.53)	0.00	50.48 (44.88)	0.001	71.39 (23.74)	0.17
Elderly people without PM	150	73.73 (17.09)		64.86 (38.64)		69.36 (22.81)		62.83 (16.68)		61.12 (17.84)		70.36 (16.27)		68.07 (35.10)		67.23 (23.76)	

EA=emotional aspect; FC=functional capacity; GHS=general health status; MH=mental health; PA=physical aspects; PM=pacemaker; SA=social aspects; VT=vitality

The two groups of elderly people showed no statistical difference regarding the physical capacity domain of SF-36 (*P*=0.17) between them. However, when analyzing each of the groups separately, there was a negative correlation between age and functional capacity for the patients with PM (*P*=0.006) and those without PM (*P*=0.009), demonstrating that functional capacity tends to decline with age.

When analyzing PCS and MCS, according to the demographic variables, there was a statistically significant difference for the variables gender, functional class, and time interval of the PM implant (Table 6).

**Table 6:** Average SF-36 physical (PCS) and mental (MCS) scores according to demographic variables for elderly patients with a pacemaker (PM).

Variables	n	PCS	*P*	MCS	*P*
		Average (SD)		Average (SD)	
**Gender**			0.021		0.005
Female	62	65.7 (22.81)		65.25 (23.74)	
Male	42	75.2 (16.75)		77.6 (18.3)	
Age (years)			0.252		0.289
60-69	43	72.37 (21.04)		74.39 (21.73)	
70-79	37	70.11 (19.11)		67.15 (24.35)	
>80	24	63.67 (21.94)		67.61 (20.30)	
**Education level**			0.508		0.958
Illiterate	17	63.69 (20.21)		68.98 (23.13)	
Elementary school	54	69.48 (19.65)		69.75 (21.33)	
High school	21	71.45 (20.19)		70.82 (25.71)	
Higher education	12	74.91 (26.79)		73.29 (23.10)	
**Comorbidities**					
Hypertension	53	67.42 (20.69)	0.283	65.72 (22.30)	0.035
Diabetes mellitus	22	65.25 (24.10)	0.588	65.54 (27.22)	0.114
**Functional classification (NYHA)**			0.00002		0.002
Class 1	28	66.77 (20.25)		64.17 (25.15)	
Class 2	6	35.04 (10.96)		46.75 (24.32)	
**Time of PM implantation (years)**			0.032		0.246
>3 months-1 year	14	69.55 (19.35)		66 (35.23)	
1-5 years	35	64.59 (19.70)		66.92 (21.78)	
>5 years	55	73.53 (21.02)		73.30 (21.71)	

NYHA=New York Heart Association

Regarding gender, men had a significantly higher average PCS and MCS than women (*P*=0.021 and *P*=0.005, respectively). Statistical differences between NYHA functional classes 1 and 2 were significant in PCS (*P*=0.00002) and MCS (*P*=0.002). Regarding the PM implant time interval, a statistically significant difference was observed only in PCS (*P*=0.032) (Table 6).

The analysis of the SF-36 physical (PCS) and mental (MCS) components average scores as well as the average EQ-5D utility score (EQoL) and the average EQ-VAS score did not show statistically significant differences among patients with and without PM (Table 7).

**Table 7:** Average scores and standard deviations of the SF-36 and EQ-5D.

Variable	n	PCS	*P*	MCS	*P*	EQoL	*P*	EQ-VAS	*P*
Elderly people with PM	104	69.56 (20.67)	0.46	70.25 (22.45)	0.15	0.729 (0.159)	0.143	70.25 (22.45)	0.152
Elderly people without PM	150	67.7 (18.76)		66.69 (16.58)		0.696 (0.186)		66.69 (16.58)	

MCS=mental component score; PCS=physical component score

## DISCUSSION

The evaluation of HRQoL is a major focus of study in medical practice, since recognizing the patient's perception of its health and disease as well as analyzing the impact of the therapeutic measures adopted is important ^[1,17]^. The use of quality of life questionnaires in patients with PM has been shown to be beneficial in evaluating the results obtained with this type of treatment ^[7,18]^. Therefore, the present study analyzed and compared, through the generic instruments SF-36 and EQ-5D, the HRQoL of a group of elderly people with PM and a group of elderly people without PM in Goiânia, Goiás.

When analyzing the influence of age on the quality of life of patients with PM, the literature presents controversial results for different age groups ^[1,19,20]^. Udo et al. ^[2]^ evaluated the quality of life of 881 patients with PM for bradycardia treatment for 7.5 years, and the worst SF-36 scores were found for those with more advanced age. However, Cesarino et al. ^[21]^, in their study on HRQoL in patients with implantable cardioverter-defibrillator, through the application of SF-36, observed that the quality of life in relation to age did not present a statistically significant difference.

In our study, we also sought to correlate the quality of life scores of the SF-36 and EQ-5D questionnaires with age; however, we did not find a significant association for the PCS, MCS, EQ-VAS, and EQoL utility scores. A similar conclusion for SF-36 was also observed by Gomes et al. ^[19]^, in which the quality of life (AQUAREL and SF-36) was evaluated after PM implantation in 23 patients. Contrary data regarding EQ-5D were seen in the study by Van Eck et al. ^[7]^ who evaluated the predictors that influenced HRQoL in 501 patients one year after PM implantation and found statistical significance when assessing age and EQ-5D utility score.

A negative correlation was observed with the age of the elderly patients with PM and those without PM only for the SF-36 functional capacity domain, demonstrating that in our study, although age cannot be considered a modifying factor of the quality of life scores in both questionnaires (SF-36 and EQ-5D), advancing age may negatively influence the individual's functional capacity, as shown by some studies in the literature ^[1,17,22]^.

Regarding the prevalence of comorbidities in elderly people with PM, 50.96% of the interviewees had hypertension (HT) and 21.15% had diabetes mellitus (DM). Similar data were found in the study by Barros ^[18]^, which evaluated the quality of life of 107 patients after six months after pacemaker implantation, in which 64.5% of the patients were diagnosed with hypertension, and 24.3% were diagnosed with DM in regular treatment.

When evaluating the gender variable, our study showed a statistically significant difference between men and women when compared to the SF-36 PCS and MCS summary measures values for the elderly with PM, in which men presented better scores in PCS and MCS than women. Similar to these findings, in the study by Udo et al. ^[2]^, lower scores were observed in the long-term SF-36 for women than for men. 

Statistical differences between genders were also significant for the mobility, usual activities, and anxiety/depression domains of the EQ-5D questionnaire as well as for the EQoL utility score and for the EQ-VAS score. In the European study by De Smedt et al. ^[23]^, a large sample of patients with stable coronary disease was evaluated according to the quality of life questionnaires (EQ-5D, SF-6D, and SF-12), and women had average EQ-5D utility score values below men, similar to the findings in our study. However, in the study by Van Eck et al. ^[7]^, there was no statistical significance when comparing participants' gender and EQ-5D domains.

In the study by Borges ^[8]^, when analyzing the influence of NYHA functional classification on the quality of life of patients with PM, a negative correlation was observed between functional class and the SF-36 PCS and MCS summary measures. Our study also showed a direct relationship between functional class and HRQoL, in which elderly patients with PM and classified as NYHA class 2 presented a significant reduction in the scores of the physical and mental components when compared to those classified as NYHA class 1. 

In the analysis of the eight domains of SF-36, patients with PM presented higher average scores in the areas of vitality, general health, and pain compared to those without PM, disagreeing with the findings of the study by Gomes et al. ^[19]^, in which patients after PM implantation showed a decrease in social and emotional aspect scores.

In the study by Cesarino et al. ^[21]^, patients with PM had a worse physical score, similar to the elderly patients with PM in our study, with an average score of 50.48, whereas for those without PM, the average score was 68.07. It should be noted, however, that the two groups are part of an age group in which the physical aspect scores may decrease with advancing age.

Statistical differences regarding PCS and MCS scores were not found when comparing the two elderly groups in this study. However, when analyzing the time variable of PM implant, for the elderly with PM, a better evaluation of PCS was observed in patients with more than five years of PM implantation. Contrary to this finding, the study by Udo et al. ^[2]^ demonstrated an increase in SF-36 scores in the short time interval after PM implantation and a gradual decline of these scores over the years.

In the study by Van Eck et al. ^[7]^, the evaluation of HRQoL in patients one year after PM implantation using the EQ-5D showed that almost 70% of the patients considered their HRQoL at least "much better" after implantation of the device. In our study, no statistical difference was found regarding the variable having PM implantation and the HRQoL evaluated using the EQ-5D. However, this study evaluated the elderly with PM for a longer period, ranging from 2 to 25 years, in contrast to the previously mentioned study, in which the implantation time considered was only one year.

Data from the literature show that, although the PM implant provides a benefit in relation to the quality of life of patients requiring this device, this improvement in HRQoL cannot be measured in older populations due to other coexisting diseases and shorter life expectancies ^[1,17,19,20]^.

## CONCLUSION

Through the evaluation of the SF-36 results, it was concluded that elderly patients with PM presented similar or greater HRQoL than patients without PM. The elderly with PM had worse scores in relation to those without PM only in the physical aspect domain of SF-36. However, it was observed in both groups that advancing age may negatively affect the individual’s functional capacity.

For the QRVS measurements of the EQ-5D, the variables gender, HT, and functional class were significant for the elderly with PM. However, the results of the EQ-5D did not show significant differences regarding the implantation of PM and HRQoL between the two groups.

**Table:** 

Authors' roles & responsibilities	
NAI	Substantial contributions to the conception or design of the work; or the acquisition, analysis and interpretation of data for the work; drafting the work or revising it critically for important intellectual content; agreement to be accountable for all aspects of the work in ensuring that issues related to the accuracy or integrity of any part of the work are appropriately investigated and resolved; final approval of the version to be published
MMN	Substantial contributions to the conception or design of the work; or the acquisition, analysis and interpretation of data for the work; drafting the work or revising it critically for important intellectual content; agreement to be accountable for all aspects of the work in ensuring that issues related to the accuracy or integrity of any part of the work are appropriately investigated and resolved; final approval of the version to be published
ASMJ	Substantial contributions to the conception or design of the work; or the acquisition, analysis and interpretation of data for the work; drafting the work or revising it critically for important intellectual content; agreement to be accountable for all aspects of the work in ensuring that issues related to the accuracy or integrity of any part of the work are appropriately investigated and resolved; final approval of the version to be published
JFF	Substantial contributions to the conception or design of the work; or the acquisition, analysis and interpretation of data for the work; drafting the work or revising it critically for important intellectual content; agreement to be accountable for all aspects of the work in ensuring that issues related to the accuracy or integrity of any part of the work are appropriately investigated and resolved; final approval of the version to be published
VAB	Substantial contributions to the conception or design of the work; or the acquisition, analysis and interpretation of data for the work; drafting the work or revising it critically for important intellectual content; agreement to be accountable for all aspects of the work in ensuring that issues related to the accuracy or integrity of any part of the work are appropriately investigated and resolved; final approval of the version to be published
